# Efforts and expectations of pregnant women against the impact of the COVID-19 pandemic: a phenomenological study

**DOI:** 10.1186/s12884-023-05383-1

**Published:** 2023-01-21

**Authors:** Arlina Dewi, Triantoro Safaria, Supriyatiningsih Supriyatiningsih, Dyah Tri Kusuma Dewi

**Affiliations:** 1 Department of Public Health, Master of Hospital Administration, Universitas Muhammadiyah Yogyakarta, Yogyakarta, Indonesia; 2 Department of Psychology, Faculty of Psychology, Ahmad Dahlan University, Yogyakarta, Indonesia; 3 Department of Obstetrics and Gynecology, Faculty of Medicine and Health Sciences, Universitas Muhammadiyah Yogyakarta, Yogyakarta, Indonesia; 4 School of Nursing, College of Nursing, Taipei Medical University, Taipei, Taiwan

**Keywords:** Anxiety, Antenatal care, Virtual based

## Abstract

**Background:**

COVID-19 is a global threat that directly impacts people’s mental health and physical well-being. This study explored the efforts and expectations of pregnant women against the impact of the COVID-19 pandemic.

**Methods:**

This study was a qualitative study that used a phenomenological approach. The informants of this study were pregnant women (*n* = 20). Data analysis used content analysis with software assistance (Nvivo Release 1.5).

**Results:**

The results of this study identified three themes which were: 1) causative factors of pregnant women’s anxiety regarding the impact of COVID-19 including lack of knowledge regarding the impact of the COVID-19 virus and perceived susceptibility; 2) Efforts to reduce anxiety during the COVID-19 pandemic including a spiritual approach, the role of family and COVID-19 prevention; and 3) Expectation regarding healthcare services during COVID-19 including virtual based Antenatal Care (ANC) Services and Private ANC Services.

**Conclusion:**

A spiritual approach, the role of family, and COVID-19 prevention will help pregnant women reduce their anxiety about being infected with the COVID-19 virus. Furthermore, virtual-based ANC Services, and private ANC services, such as home visits and dividing ANC services and general services into two different tracks as a protective mechanism from being infected with the COVID-19 virus, would assist pregnant women feel safer and secure.

## Background

COVID-19 is a global threat that directly impacts people’s physical well-being and mental health [1–3]. The World Health Organization (WHO) assesses that COVID-19 has an alarming impact due to the level of spread and severity of the illness in the community, as indicated by a tripling of cases in all countries in early 2020 and increasing further until the end of 2020 from 221 countries with 2.2% Case Fatality Rate (CFR) mortality [4–6]. In Indonesia, there were 735,124 confirmed cases of COVID-19 infection in 2020, with a death rate reaching 21,944 (CFR 3%) [5].

The increased incidence of COVID-19 infections, which seriously impact the level of illness and death, has caused community psychosocial problems, such as acute panic, anxiety, stress, obsessive behavior, paranoia, and depression [1, 7]. Data from 25 countries showed that 50.9% experienced anxiety, 57.4% experienced stress, and 58.6% experienced depression [8]. Shah et al. and Ozamiz-Etxebarria et al. explained that the increase in psychological problems during the COVID-19 pandemic was due to symptoms, comorbidities, and the quarantine period [8, 9].

A qualitative evidence synthesis, based on data from 48 studies, found that during the COVID-19 pandemic, many women experienced maternity care negatively, and that this was largely related to altered maternity care and restrictions on partner/family visiting. Some women also experienced a change in continuity of care, inconsistent care, and fewer, delayed, or cancelled antenatal and postnatal appointments, as maternity pathways were changed [10]. Moreover, the COVID-19 pandemic causes pregnant women to feel worried about being infected, and it impacts health during pregnancy for both the mother and fetus [11, 12]. A study in Canada in 2020 showed that 57% of 1987 pregnant women experienced anxiety, and 37% experienced depression. High levels of anxiety and depression in women were caused by worries about arising from the threat of COVID-19 to their lives and that of their babies [13]. In addition, 82.5% of pregnant women were worried that during labour and childbirth, the baby would become infected with COVID-19 [14]. Psychological concerns in pregnant women has deepened with the spread of information in the community, either from social media or online articles, about the high number of cases of COVID-19 infection, both among the general public and pregnant women [11, 15]. The lack of information related to COVID-19 and pregnancy has also resulted in uncertainty amongst pregnant women on the effects of COVID-19 on their health and pregnancy [15, 16].

Further, psychological problems experienced by pregnant women during the COVID-19 pandemic can result in outcomes such as psychopathology and stress that can harm the mental health of pregnant women [17]. These problems have the potential to impact on children’s cognitive and emotional development and health longer-term in the future [18, 19]. In addition, during pregnancy, psychological issues can inhibit fetal growth, premature birth, Intrauterine Fetal Death (IUFD), disability, and stunted neurodevelopment [20–22]. Thus, pregnant women must be aware of vulnerabilities to virus exposure, and stay physically and psychologically healthy, especially during the COVID-19 pandemic. This study aimed to explore pregnant women’s experience of the COVID-19 pandemic, including the impact they perceived it had on them, their efforts to reduce possible anxiety associated with potentially becoming infected with COVID-19 and their expectations regarding healthcare services for pregnant women during the global pandemic.

## Methods

### Study design

This study used a phenomenology approach, which was chosen to explore the participant’s experience of the phenomenon of interest. The phenomenology approach is a reasonable methodology for use in this study, as the study is focused on participants’ lived experience of the COVID-19 Pandemic. According to Husserl, phenomenology is defined as “the science of the essence of consciousness”. This study uses terminology and methods associated with Husserl’s phenomenological philosophy to describe an endeavor in the human sciences in which actual investigations of the psychological implications of particular experiences are conducted [23]. In the descriptive method, specific events from the everyday attitudes of others are described in detail and concretely. The descriptions are gleaned from the perspectives of involved individuals and their natural attitudes [23]. The purpose of this descriptive phenomenological study was to inquire into the perspectives and experiences of pregnant women on the COVID-19 Pandemic. For descriptive phenomenology to be successful, the researchers focus solely on the phenomenon being studied.

### Setting

This research was conducted in three provinces in Indonesia, namely the Special Region of Yogyakarta, Bangka Belitung Island, and Maluku. During the COVID-19 pandemic in the 2020–2021 period, infection cases reached 33,851 and 1155 people died in Yogyakarta, 52,359 cases and 1462 people died in Bangka Belitung Island, and 18,555 cases and 289 people died in Maluku [24].

### Study participants

In-depth interviews were conducted with 20 pregnant women in three provinces, namely the Special Region of Yogyakarta (*n* = 9), Bangka Belitung Island (*n* = 5), and Maluku (*n* = 6). Determining informants used a heterogeneous purposive sampling technique (education level, gestational age, area of residence, culture, and religion) to obtain complex diversity of information during the COVID-19 pandemic. The researchers also involved pregnant women as informants when women were willing to participate in the study. The informants were selected by midwives who were working in primary healthcare in the regions of each province. The midwives selected the informants based on purposive criteria and the willingness to be part of the study. Informed consent was taken by midwives based on pregnant women’s willingness to be part of the study. Once women indicated their willingness to take part in the study, the study researchers contacted the informants using the contact number provided to them by the recruiting midwives. The researchers, prior to interview, obtained informed consent through a Google Form. This consent also provided consent for the informants to be recorded during the interview. The researchers also utilized WhatsApp to remind the informants about their agreed online interview schedule and provided Zoom meeting/Google Meet’s links for the interviews to take place.

### Data collection

Data collection involved in-depth interviews semi-structured interviews, using an interview guide. Interviews were conducted by using either a Zoom Meeting and Google Meet platform. The researcher transcribed the recording of the in-depth interview and the transcripts were then translated to English by a linguist to ensure that meanings and narratives were not lost.

### Data analysis

This study used inductive content analysis to obtain new information or knowledge [25] from pregnant women on the COVID-19 pandemic. This study used Nvivo 1.5 software to assist the categorization and coding process based on the narratives in the transcripts [26]. This analysis method is more flexible by comparing the categorization of concepts from themes and texts in the transcript. Each researcher coded the transcripts, and all researchers came together to discuss the codes, and similar meanings, which were then classified into categories. Themes were then identified from these categories.

### Ethical consideration

The study was granted ethical approval from the health research ethics committee of Universitas ‘Aisyiyah Yogyakarta with No.1362/KEP-UNISA/I/2021. The study is also part of the Muhammadiyah Maternal and Child Center research program, Universitas Muhammadiyah Yogyakarta, to explore the impact of COVID-19 pandemic’s on pregnant women. Study participants voluntarily participated in the study and provided written informed consent prior to taking part. In addition, informants received a monetary incentive for internet package fees for the in-depth interviews. Informants received compensation for internet pulse replacement for 2 h.

### Findings

Table 1 shows that most of the informants came from urban areas (60%) in three provinces in Indonesia: the Special Region of Yogyakarta (Java Island), Bangka Belitung Island (Sumatra Island), and Maluku (Sulawesi Island). Most informants were pregnant women aged between 25 and 30 years (65%) with a college education level (70%) and were Muslim (70%). Most informants were in the second trimester of pregnancy (65%) and were a mix of primigravida and multigravida. In addition, informants had undergone an ANC examination at the clinic (75%) and/or at a PHC (52.4%).

**Table 1:** Demographic characteristics of the informants

No.	Demographic informant	***N*** = 20	(%)
1.	Geographic residence		
	Rural	8	(40%)
	Urban	12	(60%)
2.	Province		
	Special Region of Yogyakarta (Java)	9	(45%)
	Bangka Belitung Islands (Sumatra)	5	(25%)
	Maluku (Sulawesi)	6	(30%)
3.	Age of pregnant women		
	< 25 years	1	(5%)
	25–30 years	13	(65%)
	> 30 years	6	(30%)
4.	Education level		
	High school	6	(30%)
	Collage	14	(70%)
5.	Religion		
	Islam	14	(70%)
	Non-Islam	6	(30%)
6.	Parity		
	Primigravida	10	(50%)
	Multigravida	10	(50%)
7.	Gestational age		
	1st trimester	1	(5%)
	2nd trimester	13	(65%)
	3rd trimester	6	(30%)
8.	ANC check-up		
	< 4	5	(25%)
	≥4	15	(75%)
9.	Health facility		
	Clinic or Primary Health Centered (PHC)	11	(52.4%)
	Independent practice doctor	6	(28.6%)
	Hospital	4	(19%)

Three themes were identified from the collected data. These were causative factors related to pregnant women’s anxiety regarding the impact of COVID-19, pregnant women’s efforts to reduce their anxiety during the COVID-19 pandemic, and pregnant women’s expectations regarding healthcare services during the COVID-19 pandemic. Each theme consists of several categories and subcategories which are summarized in Table 2.

**Table 2:** Summary of theme analysis

NO	Themes	Categories	Subcategories
1	Causative factors of pregnant womens anxiety regarding the impact of COVID-19	Lack of knowledge regarding effects of COVID-19	• Lack of Literacy • Lack of available information.
		Perceived Susceptibility	• Being infected with the COVID-19 virus could have a serious impact on fetal • Pregnant women are more susceptible
2	Pregnant women’s efforts to reduce their anxiety during the COVID-19 pandemic	Spiritual approach	• Get closer to God • Believe in God’s Destiny
		Role of Family	• Support system • Family being understanding of their feelings
		COVID-19 Prevention	• Using social media as a direct meeting replacement • Health Status Improvement • Attend Health Protocols
3	Pregnant women’s Expectation regarding healthcare services during COVID-19 pandemic	Virtual Based of ANC Services	• Online Consultation • Online Examination Development
		Private ANC Services	• The track of ANC services and General services are divided into a different track • Home visit

### Causative factors of pregnant women’s anxiety regarding the impact of COVID-19

#### Lack of knowledge about the impact of COVID-19

##### Lack of literacy

The lack of literacy about the impact of COVID-19 has resulting in pregnant women becoming worried about their condition during the COVID-19 Pandemic.

*"I am worried and very afraid of contracting COVID-19 because I do not know the risks"* (Informant 3).

Pregnant women’s self-efficacy relies on self-care knowledge. Risk perception and pandemic awareness might also improve sensitivity. However, expectant women should be aware of the risks that could potentially make them vulnerable [27].

##### The lack of available information

The lack of available information for pregnant women regarding the impact of being infected with COVID-19 caused pregnant women to be worried about their health and the fetus.

*“My current concern is … thinking about the baby's development. Will it be affected if exposed to COVID-19?”* (Informant 2).

In social media, finding credible sources of information is crucial. While social media has been shown beneficial in educating and screening high-risk groups, the dissemination of inaccurate information can cause public fear and erode social trust. It would be advantageous to increase social trust in the information sources of medical universities [27].

#### Perceived susceptibility

##### Being infected with the COVID-19 virus could cause a serious impact on fetal

The lack of literacy about the impact of COVID-19 has resulted in pregnant women perceiving that exposure to COVID-19 in the early stages of pregnancy could be dangerous for the fetus.

*"... I am afraid... if you are exposed to COVID-19, you will miscarry because it is still in the early stages of pregnancy..."* (Informant 2).

Pregnant women did not know how COVID-19 would impact them and their baby, but what they described to know was that COVID-19 could be dangerous for them and their baby because their condition results in them being more susceptible than non-pregnant women.

##### Pregnant women are more susceptible

Pregnant women recounted that conditions during pregnancy may result in them having a higher level of susceptibility to infection from COVID-19.

*"I fear that I am afraid of getting infected because they say pregnant women are more susceptible to getting the virus... because their immune system is lower..."* (Informant 1).

*"I am afraid because pregnant women are more vulnerable, while I do not eat enough … lately, that is what I think"* (Informant 4).

Perceived susceptibility of pregnant women caused by their beliefs that a pregnant woman has a low immune system. Some of them believed that hormonal change which affected their appetite causing them to eat less could reduce their immunity, and thus increase their risk of being infected with COVID-19.

### Pregnant Women’s efforts to reduce anxiety during the COVID-19 pandemic

#### Spiritual approach

##### Get closer to god

The pregnant women in this study believed that getting closer to God was one way to reduce their anxiety related to being infected with COVID-19. They could share their worries to God and pray to God not to be infected with COVID-19. Praying to God made them feel more relieved in being able to face the COVID-19 Pandemic.

*"I happen to be the type of person who gets stressed easily because of paranoia … thus, I pray more to God even if it is only in my heart, like, oh God, I hope this COVID-19 problem does not come until us..."* (Informant 6)

##### Believe in God’s Destiny

Believe that God will always give His help to everyone who’s praying to Him calmed pregnant women’s hearts and minds and reduced their worry about being exposed to COVID-19. Also, thinking positively that whatever happens is God’s will helped reduce their worries about being infected with COVID-19. As one pregnant mother said

*: "... Only strengthened by praying so that worries go away. Thus, thinking now that it is Covid … you have to pray if you are worried … it feels like you want to be carried away by the burden of your mind, so pray, and it is impossible for God not to want to help … there is still God. Hence, leave everything to God for whatever happens to us … .”* (Informant 7)

#### Role of family

##### Support system

Family support could provide a sense of security and comfort for pregnant women. Family support gave pregnant women strength to face the pandemic, because they feel cared for and guarded by their family, which made them feel safer from being infected with COVID-19.

*“It is a form of support for my family … Mom said, "Take care of your health, do not think about it too much … if you want to leave the house, you have to wear a mask.” They say to take care of your immunity because it is easy to get infected when pregnant. If the support from your husband's family says take care of your health, hopefully, during this pandemic, the delivery will be smooth without any disturbances … ."* (Informant 3)*.*

During the COVID-19 pandemic, the support of family members is an essential component for maintaining the mental health of pregnant women. Enhancing the support that pregnant women receive from their families is one way to assist enhance their mental health [28].

##### Family being understanding of their feelings

Family or husband understanding pregnant women’s feelings by giving attention also provides a sense of security and comfort for pregnant women.

*“The most emotional support … , I feel like this … the feeling is like this, most husbands say calm down, especially during the time of covid so paranoia … so paranoid... he understands because he understands more (health workers) than we ordinary people. He explained well … do not worry. Hence, I calmed down a bit. He said the covid problem was okay, and the important thing was to be diligent in wearing masks because all of them were from droplets … so do not worry. Then, he also said to keep the condition with food control do not forget to take vitamins. If there are many or few covid patients, he will not say anything because he knows I am a panicky person … that is so paranoid … .”* (Informant 6)

#### COVID-19 prevention

##### Using social media as a direct meeting replacement

The COVID-19 pandemic caused pregnant women to restrict their lives in the outside environment. Pregnant women also preferred to use social media for communicating, shopping, working and gathering to reduce the contact with people outside. The convenience of industry 4.0 in activities, communication, and social relations can support pregnant women in reducing contamination with the outside environment. As two pregnant women recounted:

*“ … for work problems that are urgent or arguably not urgent, we can meet via zoom or maybe a conversation meeting via google meet and WhatsApp, video calls … be diverted there. However, some things must be spoken face-to-face or be done at work, of course trying to get to work … ."* (Informant 2).

*“Now I have been looking for information and preparing (buy) baby clothes; I worried (about being infected) if go outside it feels like I will die, (so) I just look for the baby clothes (through) online (marketplace) … .”* (Informant 10)

Everything is going to be easy as long as there is an online media. It is utilized by pregnant women to reduce their outside activities. The pregnant women feel safer because they did not to go outside, they believed that they would not be infected with COVID-19 as long as they did not meet other people.

##### Health status improvement

Pregnant women believed that meeting nutritional needs during pregnancy was the right way to maintain a healthy body.

*"I am afraid … if pregnant women are vulnerable … do not eat enough. Oh, I have to eat because there is a covid pandemic.... must eat a lot … must eat fruit … "* (Informant 4)*.*

Women in the study believed that a lack of nutrition could affect their immunity, therefore some women tried to increase their nutrition and eat healthy food in as far as they could.

##### Attend to health protocols

Pregnant women applied health protocols when leaving the house, such as using masks, and hand sanitizer, washing hands, and reducing touching public items.

*"When you leave the house, you usually use a mask … the hand sanitizer is in your bag, you immediately wash your hands or spray the hand sanitizer until your pants and the back of the motorbike are sprayed ..."* (Informant 15)*.*

*"Back again, because of need, follow the health protocol and do not touch carelessly... must wear a mask..."* (Informant 11).

Pregnant women believed that applying health protocols when outside and arriving at home was the right way to prevent the transmission of COVID-19.

### Pregnant women’s expectations regarding healthcare services during COVID-19

#### Virtual-based ANC services

##### Online consultation

ANC services with online media can also be carried out specifically for consulting or inspection services that do not require direct action.

*“Now it is easy … maybe you can go online … . You can do online consultations, but maybe you cannot go straight to USG. Maybe go online for consultation maybe you can complain … ”* (Informant 8).

Pregnancy-specific stress may be exacerbated by a lack of access to antenatal care as a result of limitations put in place in reaction to the COVID-19 pandemic [16]. Pregnant women thought that online consultation would make ANC services become easier and safer during COVID-19 pandemic. They only needed to access care through an application such as telehealth to interact with healthcare providers.

##### Online examination

Online examinations can be a breakthrough in the implementation of ANC so that the involvement of health workers and pregnant women is not interrupted. As one pregnant woman said:

*“ … . maybe later, it can be developed for online examination, although we need to check directly, for several times, it may be necessary to go online instead of directly checking at the place (clinic). Because if I go to the clinic often … I check once a month until I am confused about what else to ask. I feel like I just asked this last month … this again … but I need that too, so if there are complaints like that … you can go online … .”* (Informant 16).

Virtual-based ANC services can provide convenience and comfort for pregnant women because it reduced the need for direct contact during the COVID-19 pandemic. The service was also available in real time without waiting for a scheduled visit. One pregnant woman said:

*" … that is good. There is no need to go out there; you do not have to face it … it is more real-time. You do not have to go to the clinic. If you are pregnant, you have to have regular check-ups, and you will continue to meet with the doctor, but if there is a virtual, it is safer … ."* (Informant 13).

#### Private antenatal care flow in health facilities and the community

##### ANC services and general services are divided into different tracks

The fear of being exposed to COVID-19 resulted in pregnant women expecting that the flow of ANC services from registration to the examination must be differentiated to ensure the health of pregnant women during the examination process.

“ *… the hope is that the place to check for pregnant women … the route to the examination is different, the path for pregnant women who want to be examined in that way. If this cannot be distinguished … the hallways are still together; only the rooms are different, and the queues, the waiting areas, and the prayer rooms are all still together. We hope that everything is devoted to pregnant and lactating mothers; we want our path to go to a different place, especially for pregnant people, then we make sure that all pregnant is healthy from Covid”* (Informant 16)*.*

In order to reduce crowding, some health facilities stipulated that only pregnant women could access services from registration to ANC examination.

*"... no one can accompany, the accompany guy is waiting outside... the ultrasound results are only allowed to be video, but if you go to the hospital, you cannot... only patients can enter"* (Informant 9).

##### Home visit

The pregnant women also have an expectation that ANC services could have empowered health cadres to take home visit monitoring to minimize COVID-19 transmission in health facilities.

*"It is better if, for example, there are village cadres from the PHC who go to the houses of pregnant women than we have to gather in one place (*posyandu*)... we become victims (exposure to COVID-19) ..." (*Informant 6).

## Discussion

This study revealed that pregnant women experienced anxiety during the COVID-19 pandemic and a lack of knowledge regarding the impact of COVID-19 on pregnancy and childbirth. Inadequate knowledge by pregnant women was related to the risk of exposure to COVID-19 and vertical transmission resulting in negative attitudes toward preventing COVID-19 transmission [29, 30]. Pregnant women’s knowledge was mostly obtained from social media information, which is known to have less accuracy than information from health workers, newspapers, and the WHO or CDC website [29]. Rumors, stigma, and conspiracies can affect pregnant women’s trust in misinformation and disinformation. The selection of information must also be based on accurate and reliable sources so that health agencies and government stakeholders can track misinformation in real-time [31, 32]. Improving the literacy of pregnant women during the COVID-19 pandemic can help strive for prevention to strengthen the involvement of pregnant women in making decisions and empowering physical and psychological health [33].

Pregnant women practiced self-efficacy through internal and external approaches. Psychologically, pregnant women took an internal approach by getting closer to God and thinking positively during the pandemic and an external approach through family support. Generally, health workers (56%) did not discuss spiritual context when caring for patients with anxiety [34]. Evans et al. also mentioned that midwives only provided treatment for pregnant women with anxiety through support, attention, and relaxation skills [35]. A psychological approach through spirituality in handling individual anxiety related to belief in God, which is practiced in daily activities such as prayer, worship, and believing in God’s commands, can foster hope, encouragement, and positive attitudes towards health problems. One study found that 91.7% of pregnant women who read the Qur’an experienced decreased anxiety [36]. Curlin et al. added that strong spirituality impacted patients to cope with health problems [34]. An external psychological approach showed that family support provided pregnant women with a sense of security and comfort during the COVID-19 pandemic. Tang et al. explained that low support from family and husband was one of the risk factors of anxiety in pregnant women, with a prevalence of 91.86% [37]. Commonly, social and family support, also affected self-efficacy and the welfare of pregnant women [38]. Yue et al. explained that family and partner support is crucial in reducing the adverse effects of pressure felt by pregnant women during the COVID-19 pandemic. Positive reinforcement could also provide an emotional experience [39].

Physically, pregnant women took internal and external approaches to prevent the transmission of COVID-19. This study showed that pregnant women avoided exposure to COVID-19 through an internal approach by implementing strict health protocols using online media to reduce direct interactions. Ames et al. explained the high cases of pregnant women with COVID-19 infection because they were unaware of their contact with the COVID-19 sufferer (76.8%) [40]. Pregnant women were also unaware of their condition because they did not show symptoms of exposure to COVID-19, so it was too late to take proper infection prevention and control [41].

Efforts to prevent COVID-19 transmission in the community can be carried out by taking self-limiting steps in social interaction, limiting activities, and implementing health protocols (washing hands and wearing masks) [42]. Implementing social distancing and health protocols can reduce the spread of respiratory viral infections during the COVID-19 pandemic [43]. Sikali’s research found that social distancing has a negative impact, such as growing impersonality and individualism and loss of sense of community because of social reception [44]. The negative impact of social distancing could be minimized by using social media to replace direct interactions. Online interactions can also be implemented to maintain good social relations and bring the family closer and wider connections in the community [45–47].

Despite efforts of pregnant women to prevent COVID-19 transmission, women expect the government to guarantee their health by changing the antenatal care services system, both at the health facility level and in the community. WHO has urged restrictions on providing health services and postponement of care for pregnant women as needed [48, 49]. Health monitoring does not run as before the pandemic since the intensity of meetings with health workers is reduced [42, 50]. Restrictions for pregnant women in accessing health facilities should be replaced by optimizing virtual services that impact patient satisfaction [51–53]. Telehealth services are an alternative for pregnant women to reduce their exposure to COVID-19 during prenatal care [54]. Based on the findings, pregnant women expected a change in the flow of ANC services privately in health facilities and the community. The role of health cadres is to make a home visit, so they do not need to visit health facilities.

The limitation of this study is that data collection was undertaken by using Zoom and Google Meet. An unstable signal during the in-depth-interview process caused rendered researchers unable to hear some information clearly, so the researchers needed to reconfirm the statements.

## Conclusion

Pregnant women feel that their condition is more susceptible to being infected with the COVID-19 virus than the general population, which caused the pregnant women to experience anxiety about being infected with COVID-19. Nevertheless, pregnant women experienced that efforts such as adopting a spiritual approach, the role of family, and COVID-19 prevention helped them to reduce their anxiety about being infected with the COVID-19 virus. The pregnant women also expected that healthcare services would provide virtual based ANC Services and private ANC services such as home visits and divide ANC services and general services into two different tracks to protect them from being infected with the COVID-19 virus; this would help the pregnant women feel safer and secure.

## Data Availability

The datasets used and/or analyzed during the current study available from the corresponding author on reasonable request.

## Abbreviations

WHO: World Health Organization
CFR: Case Fatality Rate
CDC: Centers for Disease Control and Prevention
IUFD: Intrauterine Fetal Death
PHC: Primary Health Centered
